# Societal determinants of HIV vulnerability among clients of female commercial sex workers in Indonesia

**DOI:** 10.1371/journal.pone.0207647

**Published:** 2018-11-21

**Authors:** Nelsensius Klau Fauk, Christina Yeni Kustanti, Ririn Wulandari, Ayi Diah Damayani, Lillian Mwanri

**Affiliations:** 1 Institute of Resource Governance and Social Change, Kupang, Nusa Tenggara Timur, Indonesia; 2 College of Medicine and Public Health, Flinders University, Adelaide, South Australia, Australia; 3 Bethesda Yakkum Health Science Institute, Kota Yogyakarta, Daerah Istimewa Yogyakarta, Indonesia; 4 Public Health Faculty, Malahayati University, Kemiling, Bandar Lampung, Indonesia; 5 Midwifery Department, Poltekkes Pangkal Pinang, Bangka Tengah, Indonesia; University of South Florida, UNITED STATES

## Abstract

This study aimed to explore societal determinants of HIV vulnerability among the clients of female commercial sex workers (FCSWs) in Belu and Malaka districts, Indonesia. A qualitative inquiry using in-depth interviews was employed to collect data from participants (n = 42) recruited using a purposive and snowball sampling technique. Data analysis was guided by a qualitative data analysis framework. The study results revealed several societal determinants that supported vulnerability to HIV infection among the participants. They included low education level and sexual health literacy including the lack of knowledge and information about HIV transmission and prevention. Additional determinants identified were limited source of HIV/AIDS-related information, availability of and ease of accessibility of brothels and FCSWs, peer influence, and high mobility of the study participants. Findings of this study indicate the needs and call for interventions that aim to protect both FCSWs and their clients, through provision of HIV/AIDS and sexual health education and information and improvement in the availability and accessibility of condoms.

## Introduction

The prevalence of HIV infection among the clients of female commercial sex workers (FCSWs) and the sex workers themselves has been reported to be ranging from 5.6% to 34.9% in multiple global studies [1–8]. The World Health Organisation [9, 10] has therefore identified the clients of FCSWs as well as the sex workers as one of the groups at higher risk for HIV infection. However, this group does not seem to be considered as an HIV high-risk group and has not been the target group for HIV prevention programs and interventions in Indonesia even though the number of annual HIV cases transmitted through heterosexual contacts is higher than that of through other modes of transmission [11]. For instance, heterosexual contacts have been reported as the main route of HIV transmission as they contributed 36.05% of the total of 182,233 HIV cases in the last five years [11]. It is consistently reported that there have been an increase in HIV number leading to an accumulation of 398,094 HIV/AIDS cases in Indonesia [11].

Despite the upward trend of HIV transmission through heterosexual contacts, evidence on the determinants of HIV vulnerability among the clients of FCSWs is scarce in Indonesia. In the context of this paper, vulnerability determinants refer to a range of contextual, socio-environmental and cultural factors that contribute to the spread of HIV and are instrumental in enhancing the transmissibility of the HIV infection among clients of FCSWs and in other vulnerable populations. These factors may include: health literacy levels, ineffective dissemination of HIV/AIDS information, social interactions and relationships, mobility factors, and availability and ease of accessibility of brothels and FCSWs [12–15]. Societal determinants on the other hand, refer to a much broader range of social factors (distal factors) that are supportive of HIV transmission in the current study group as well as in the general society: these may include factors such as education levels, socioeconomic status, the prevalence of HIV and poorly resourced health system that does not meet the needs of the population especially those at highest risk of HIV infections [16–19].

Globally, behavioural factors such as frequent unprotected sex with multiple sex workers, expectation of high sexual pleasure and sexual satisfaction have been reported to be the main contributors (proximal factors) to the transmission of the epidemic among a group of people who are the clients of FCSWs [1, 3, 4, 6, 7, 19–27]. Lack of knowledge of how HIV transmission and prevention can occur, the practice of consuming prophylactic antibiotics prior to sexual engagement as an HIV prevention method and perceptions of good appearance as HIV safe sex partners, have also been identified as influencers of unsafe sexual practices among the clients of FCSWs [4, 6, 26–29]. Likewise, trusting steady FCSWs is also associated with decreased condom use among them [26]. Other factors reported to enhance inconsistent or non-condom use among study participants leading to increased vulnerability to HIV transmission include unavailability of condoms immediately before the sexual act, alcohol and drug use and the belief that condoms are not effective in preventing HIV transmission [4, 6, 7, 28].

The results of previous studies as reported above indicate a lack of evidence on the social factors that contribute to shaping the HIV vulnerability among the clients of FCSWs globally and in the context of Indonesia in particular. This study aimed to explore societal determinants of HIV vulnerability among the clients of FCSWs in Indonesia.

## Methods

The consolidated criteria for reporting qualitative studies (COREQ) checklist (S1 Fig) was used to guide the report of the methods section [30]. The 32 items COREQ checklist helped the researchers to ensure that the reporting of this qualitative study is explicit and comprehensive [30].

### Conceptual framework

The current study employed the social determinants of health framework [31] to guide the data analysis. This framework recognises the pivotal role that social factors (economic, environmental and cultural) play in increasing the vulnerability for a health condition to a population living with vulnerability. The influence of these factors is not dissimilar to HIV infection among the current study participants. Additionally, recent studies have emphasised individual-level determinants, including inconsistent and lack of condom use or risky sexual behaviours and multiple number of sex partners as key vulnerability factors for HIV transmission among clients of FCSWs [1, 3, 7, 20–23, 26]. Increasing HIV/AIDS prevalence and poor knowledge about STI in Indonesia have also been attributed to the HIV transmission among clients of FCSWs [25, 28, 29]. The social determinant theoretical framework assumed in this study considers the multi-causal factors attributable to HIV transmission in this group.

### Study settings

Belu and Malaka districts were one district called Belu prior to the separation in 2012. They are located in East Nusa Tenggara province, the Eastern part of Indonesia. Belu district shares a border with East Timor in the East, *Timor Tengah Utara* district in the West and Malaka district in the South. Malaka district shares the border with East Timor in the East, *Timor Tengah Utara* and *Timor Tengah Selatan* districts in the West, and Belu district in the North. Belu and Malaka districts respectively cover the area of 1,284,94 km^2^ and 1,160,63 km^2^, with the total population of 204,541 people including 100,922 male and 103,619 female, and 171,079 people comprising 83,492 male and 87,587 female [32, 33]. Each district comprises 12 sub-districts. Health facilities in Belu district are 3 hospitals, 17 community health centres, 21 sub-community health centres, 48 village maternity posts, 23 village health posts and 5 private clinics. Health facilities in Malaka district include 1 hospital, 17 community health centres, 25 sub-community health centres, 5 private clinics, and 82 village health posts or village maternity posts [32–34].

The available data show that the total number of HIV/AIDS cases in the two districts is 921, and unprotected sexual behaviour is the main mode of transmission [35]. Although FCSWs and their clients as previously reported in Indonesia and globally are among the groups at high-risk for HIV infection [2, 3, 7, 25, 27, 29], information about the prevalence of the epidemic among FCSWs and their clients in the study settings and in Indonesia as whole is not available. However, some previous studies with motorcycle taxi drivers in the current study settings have reported unprotected sexual behaviour with multiple FCSW partners as the main contributor for HIV transmission among them [19, 36].

### Data collection and procedure

A qualitative inquiry using in-depth one-on-one interviews was employed to explore societal determinants of HIV vulnerability among the clients of FCSWs. The interviews were conducted by the first three authors (NKF, CYK and RW). NKF was a full-time researcher at a research institution in Indonesia and had been involved in studies on HIV/AIDS topics with different population groups. He is a trained researcher with three master’s degrees in the field of HIV/AIDS, health education and promotion, and health and development. CYK was a lecturer and researcher at an academic institution in Indonesia. She had attended different study courses and had two master’s degrees in the field of nursing and palliative care. RW worked as a lecturer and researcher. She had a master’s degree in the field of public health. The interview guide focused on several key areas including the education level, HIV/AIDS-related health literacy, sources of HIV/AIDS-related information, mobility, social relationships among the clients of FCSWs and between them and FCSWs, social influence among the clients of FCSWs, the environment where they lived, worked and interacted, the availability and accessibility of brothels and FCSWs. A purposive and snowball sampling technique was used to recruit the study participants. Inclusion criteria for the study participant were (a) individuals who aged 18 years old or above, and (b) a male client of FCSWs. Participants were recruited in three waves as follows: A known staff member of a non-governmental organisation (NGO) that provides HIV/AIDS services in the districts was asked to help disseminate study package containing the study information and researchers’ contacts to the clients of this NGO. The study package described the aim of the study and asked the readers to contact the researchers should they want to participants. Four initial potential participants known to be clients of FCSWs were provided with the study package and all of them contacted the researchers, consented and formed the first wave of the interviews. These participants were also asked to disseminate the study package to further potential participants known to them. Eighteen participants contacted the researchers, consented and became the second wave of interviews. Similarly, these participants were asked the same and twenty participants took part in the third wave of interviews. None of the potential participants who stated their willingness to participate withdrew their participation prior to or during the interviews. A total of 42 participants were interviewed to reach data saturation [37] once we felt that the information or responses provided by the last few participants who had been interviewed were similar to those of previous participants. At the end of the interviews, we asked the participants whether they would like to read a copy of each interview and edit it prior to us analysing it but none of them requested to see it. No repeat interviews were conducted. There was no any relationship established between the researchers and any of the participants prior to the study.

Interviews took place at convenient times and places recommended by each participant. The majority of the interviews took place at the houses of the participants and a few other were conducted at the house of the first author/interviewer (NKF) and the workplaces of the participants. No one other than the interviewers and participants was present during the interviews. Prior to each interview, participants got a brief explanation about the backgrounds of the researchers and were again reminded about the purpose of the study. Participants were also informed that: (i) interviews would take approximately 45 to 60 minutes and be recorded using a tape recorder, and notes would be taken by interviewers during the interviews, (ii) their participation was voluntary and they could withdraw any time when they wished to do so, (iii) there would be no individual benefits or consequences to participate or not to participate, (iv) data or information they provide during the interview would be treated with strict confidentiality and (v) data or information would be deidentified and not linking back to any individual—only a number and a letter (e.g. p1, p2, . . . .) would be assigned to each participant. Before the interviews, participants were informed that ethics approval for this study was obtained from Medicine Research Ethics Committee, Duta Wacana Christian University, Indonesia (ref: 386/C.16/FK/2017). Each participant was provided with a written informed consent, signed and returned it at the interview day. Each interview was conducted at a time and place recommended by each participant.

### Data analysis

Data were transcribed verbatim and translated into English and crossed check for accuracy, clarity and the quality of translation by three authors (NKF, CYK and RW). This was conducted to maintain the reliability and validity of the collected data. These authors are fluent in Bahasa and English. Data were analysed using the framework analysis by Ritchie and Spencer for qualitative data [38]. The framework analysis provides a systematic approach to the management of qualitative data in a coherent and structured way, and enhances rigour, transparency and validity of the analytic process. The framework analysis involves five steps including: (i) *familiarisation* with the data or transcripts by repeatedly reading through and breaking down the data into small chunks of data while commenting or labelling, (ii) *identifying a thematic framework* through writing down recurrent key issues and concepts, (iii) *indexing* the *data* by creating a list of codes (open coding) to identify similar codes or redundant codes to reduce a long list of codes to a manageable number. Closed coding was conducted afterwards to group similar codes under the same theme. The themes included educational level and sexual health literacy, availability of brothels and FCSWs, impact of peer influence, and influence of mobility on HIV transmission, (iv) *charting* of data by arranging appropriate thematic references in a summary of chart which helped the researchers to make data comparisons across interviews and within each interview, and (v) *mapping and interpretation of the data where data* examination and interpretations were carried out [38, 39].

## Results

### Profile of participants

The median age of the study participants was 27 years and interquartile rage (IQR) was 7. The majority of the participants graduated from high school (see Table 1). Lack of condom use and inconsistent condom use when having sexual encounters with FCSWs during the past six months were reported high among the participants. The participants worked as motorcycle taxi drivers, construction workers, and port workforce (loading and unloading materials), while a few were unemployed.

**Table 1:** Participants’ demographic characteristics.

Characteristics	No. of Respondents N = 42 (%)
*Age*	
19–25	16 (38)
26–30	18 (43)
31–35	8 (19)
*Condom use with FCSWs in past 6 months*	
Never	18 (43)
Sometimes	16 (38)
Always	8 (19)
*Number of sexual encounters with FCSWs in the past 6 months*	
1–5 times	5 (12)
6–10 times	11 (26)
≥ 11 times	26 (62)
*Education*	
High school graduates	35(83)
Elementary school graduates	7(17)
*Condom use with FCSWs in past 6 months*	
Never	18 (43)
Sometimes	16 (38)
Always	8 (19)
*Occupation*	
*Ojek* (motorcycle taxi drivers)	15 (36)
Construction workers	14 (33)
Harbour workers	10 (24)
Unemployed	3 (7)

### Educational level and sexual health literacy

Low level of education seemed to be one of the social determinants supportive of HIV vulnerability among the study participants. This was reflected in the fact that the majority of the participants (n = 24) lacked information and knowledge of HIV/AIDS especially the means of HIV transmission and prevention. This led to their consistent engagement in unprotected sexual behaviour with multiple FCSWs without realising the increased vulnerability to HIV as well as other sexually transmitted infections (STIs) transmission:

“I never learned anything about HIV/AIDS. I quitted school after graduating from elementary school, so I do not get in touch with information related to sexual health or HIV/AIDS in particular. . . . . I do not know about condoms, . . . . I do not use condoms” (P7).

“When I was at senior high school, my Biology teacher once introduced us the names of diseases including HIV/AIDS but there was not any further explanation about how people contract HIV/AIDS” (P13).

“I heard some friends of mine mentioned about HIV/AIDS and condoms, but I do not understand at all. . . . . I never use condoms . . . .” (P32).

Limited source of HIV/AIDS information was also indicated to contribute to shaping the HIV vulnerability among the clients of FCSWs. Friends and HIV/AIDS sessions held by the local AIDS Commission were mentioned by eight participants who knew about HIV/AIDS and consistently used condoms as the only sources of information about HIV/AIDS:

“I firstly heard of HIV/AIDS from a friend of mine and then I also attended the socialisation of HIV/AIDS information held by the [local] AIDS commission” (P1).

“I know a little bit about HIV/AIDS because I participated in the HIV/AIDS session in my sub-district. The staff from AIDS Commission explained about how the infection spreads including through unsafe sexual contacts. They also talked about condoms as a means of HIV prevention” (P9).

“. . . . I have a friend who works for a nongovernmental organisation that provides HIV/AIDS services. He told me about HIV/AIDS and convinced me to use condoms to protect myself from HIV/AIDS and syphilis or gonorrhoea” (P42).

### Availability of brothels and FCSWs

The availability of brothels and FCSWs within vicinity of their residence was found to be a supportive environment for HIV vulnerability among the clients of FCSWs. Ease of access to FCSWs for sexual activities, compounded by unavailability of condoms when urgently needed and inconsistent condom use reported by sixteen participants heightened the participants’ vulnerability to contracting HIV infection:

“There are a few brothels here, so if I want [to have sex with sex workers] then I can just go there . . . . about condom use, it is up to me, sometimes I use but sometimes I do not” (P16).

“Here the sex workers are everywhere and there are always new faces, mostly coming from Java. So, it is easy to find them. . . . . I do not use condoms that often because they [FCSWs] do not provide condoms and do not complain either if I do not use” (P23).

“. . . . After my first visit [to a brothel] I feel tempted to go there if I get a chance . . . . most of the time I do not use condoms because I do not have” (P36).

Some participants had developed good relationships with FCSWs. These relationships facilitated the ease and frequency of engaging in unsafe sex. Seven participants acknowledged to have unprotected sexual intercourse with multiple FCSW partners because they knew and trusted them or were in a relationship. These interactions enhanced the likelihood of HIV transmission among them:

“I have visited them [FCSWs] a few times and got to know some of them. Sometimes we text each other . . . . if I have sex with the ones [FCSWs] I know I do not think of using condoms . . . .” (P41).

“Three of them [FCSWs] are my friends, I mean we know each other because I had sex with each of them a few times. I have their mobile phone numbers, so I can contact any of them anytime if I want to have sex. . . . . I do not use condoms once having sex with any of these three because we know each other, and I trust them. I think they would tell me they have problems like infections” (P24).

“They [FCSWs] often come to the harbour to find clients in the evening. I know a few of them and have done it [have sex] several times with them. . . . . I do not use condoms because I know them, and I do not have condoms either, but sometimes I use [condoms]. . . .” (P16).

### Impact of peer influence

The social influence among peers of the clients of FCSW was indicated to be one of the important determinants contributing to HIV vulnerability among the study participants. Eleven participants articulated instances that they were influenced by their peers which led to not using condoms when they had sex with FCSWs. These were the social conditions supportive of HIV and other STI transmissions among male clients of FCSWs:

“I sometimes visit the brothel alone, but often my colleagues and I make an agreement and go there together” (P20).

“. . . . sometimes my friends call and ask me to go with them if they have a plan to visit the sex workers. . . .” (P35).

“I often use condom but if I go together with my colleagues [to visit FCSWs] and one of us does not have it [condom] then we all often decide not to use it. It sounds funny but it has happened a few times. . . . .” (P4).

“I once talked to a friend of mine about condoms and he told me that it does not feel good during the sexual intercourse if using condoms. . . . . I never used [condoms]” (P3).

### Influence of mobility on HIV transmission

High mobility was also discerned to be a condition that played significant influence in shaping sexual behaviours of the clients of FCSWs, hence enhancing HIV vulnerability among them. Nineteen respondents expressed that frequent work-related mobility from one place to another increased the opportunity for sexual encounters with multiple FCSWs:

“I often move from one place to another for a short term of one to three months. My colleagues and I work on constructions such as houses or school buildings. . . . . I think that is the why I frequently have sex with different sex partners [FCSWs], I often meet new partners in new places” (P27).

“. . . . Mostly they [FCSWs] are in the markets or shopping centres or in front of some hotels in the evening. As a motorcycle taxi driver, I spend most of my time driving around the town, so I can easily recognise them. . . . . Sometimes I bargain and if she agrees on the price then I take her to my accommodation and have sex” (P34).

“I go to the harbour every day during the day time or in the evening and often meet sex workers over there. They come to the harbour in the evening to look for clients, . . . . sometimes I do not plan [to have sex with sex workers] but the desire often arises once I see them, so it [sexual intercourse] happens” (P39).

Furthermore, individuals who frequently moved from one place to another due to work were also prone to inconsistent use of condoms. Unavailability of condoms, inability to access condoms (due to lack of knowledge of where they are sold and cultural norms where people are not accustomed to carrying condoms), were additional factors supportive of unprotected sexual encounters with FCSWs among fourteen interviewees:

“. . . . It depends, if it [condom] is available then I use, if it is not then I do not use. . . .” (P34).

“. . . . I do not use condoms. Condoms are not available at the harbour or nearby stores” (P17).

“I often do not use condoms because I do not know where to buy them, especially in the new places I just moved into” (P12).

“I do not use condoms when having sex with sex workers I meet somewhere while driving because I am not accustomed to bringing condoms with me when I go to work or drive” (P2).

## Discussion

The World Health Organisation has reported clients of sex workers to be a group at a higher risk for HIV infection [10]. Their frequent engagement in unsafe sex with multiple sex workers has been incriminated as one of the main factors that increases the possibility of HIV and other STIs acquisition among them [1, 3, 4, 6, 7, 19–29, 40]. Consistent with the results of the studies elsewhere [4, 6, 19, 26–29, 36], the findings of this study confirm that low level of education was an important social determinant that contributed to shaping the HIV vulnerability among the clients of FCSWs. This was reflected in the fact that the majority of the study participants had low level of education or lacked knowledge and information on how HIV transmission and prevention could occur. This was reported to be a strong factor supportive of inconsistent or lack of condom use, hence increasing the HIV vulnerability among them. The current study also reports limited sources and/or lack of dissemination of HIV/AIDS-related information within the environment where they lived. The lack of resources and limited information were parts of the social conditions that played a role in shaping unsafe sexual behaviours escalating HIV vulnerability among the study participants.

Availability of brothels within communities or proximal to participants’ residences led to ease of access to sex workers. The ease of access was also compounded by unavailability of or poor access to condoms. These were complex socio-environmental conditions that enhanced engagement of male clients in HIV-risk sexual encounters with FCSWs. This study also suggests that there was establishment of trusting sexual relationships between participants and sex workers. These relationships led to clients not using condoms due to mutual trust that developed between these clients and their female sex service providers. These behaviours are not uncommon as trusting relationships have been stated to be very strong as partners develop mutual confidence that neither of them abuses the other’s vulnerability [41]. These findings also support previous studies [12, 19, 26, 36] reporting trust in steady FCSWs and sexual relationship as determinants of unprotected sexual behaviours that supported the HIV transmission among the clients of sex workers and motorcycle taxi drivers. Other factors such as sex workers dislike of the use of condoms, condoms bursting during the sexual act that have been reported in previous studies’ results [26, 29] were not among the findings of the current study.

The peer influences among clients of FCSWs that encouraged frequent engagement in sexual intercourses with sex workers and inconsistent condom use were also identified as underpinning societal determinants of HIV vulnerability among the participants. Similar findings have been reported in previous studies with motorcycle taxi drivers and men who have sex with men [12, 19]. This indicates that peer influences particularly through sharing of information about the availability of FCSWs and linking up each other to FCSWs increased the frequency of their engagement in unsafe sexual intercourses with sex workers. The contribution of mobility as a driver to spreading HIV infections among other population groups and communities has been reported in several previous studies [19, 42–45]. This was also detected in the current study, suggesting that being highly mobile and moving from one place to another for short-term stay for work purposes increased the study participants’ probability of unsafe sexual encounters with multiple FCSWs.

The study cannot be complete without pointing out its limitations. The study covered only a limited number of participants with similar characteristics in terms of working backgrounds, levels of education, and patterns of mobility. This could have led to biased overview of societal determinants supportive of HIV vulnerability among the clients of FCSWs. The results are therefore less likely to be generalised to other clients of FCSWs with different characteristics and in different settings in Indonesia and globally. However, the findings of this study can be used to inform future studies to cover heterogeneous participants with different backgrounds in terms of education levels and work, and from different settings.

## Conclusions

The findings of the current study report several potential societal determinants that may contribute to shaping HIV vulnerability among the clients of FCSWs. They include low education level and sexual health literacy, limited and/or lack of knowledge and information on how HIV transmission and prevention occur, availability of and easily accessible brothels with attractive FCSWs, ease of mobility and peer influence among the study participants. These factors play important roles in supporting frequent engagement of the clients of FCSWS in unprotected sexual activities with multiple sex workers, hence increasing the HIV vulnerability within this group. Findings of this study indicate the need for HIV/AIDS awareness raising interventions that would cover both the FCSWs and their clients and provide HIV/AIDS and sexual health education and information. There is also a need to improve the availability and accessibility of condoms for both FCSWs and their clients. Further studies are recommended to understand what could be done by governmental and non-governmental institutions to prevent and reduce HIV/AIDS problem among these population groups.

## Supporting information

S1 Fig COREQ Checklist. (DOCX)
